# Comparative Analysis of Polyphenolic Compounds in Different *Amaranthus* Species: Influence of Genotypes and Harvesting Year

**DOI:** 10.3390/antiox13040501

**Published:** 2024-04-22

**Authors:** Jun-Hyoung Bang, Ick-Hyun Jo, Raveendar Sebastin, Won Tea Jeong, Sangtaek Oh, Tae-Young Heo, Jeehye Sung, Tae kyung Hyun, Yoon-Sup So, Ju-Kyung Yu, Amal Mohamed AlGarawi, Ashraf Atef Hatamleh, Gi-Ho Sung, Jong-Wook Chung

**Affiliations:** 1 Department of Industrial Plant Science and Technology, Chungbuk National University, Cheongju 28644, Republic of Korea; peerage8794@gmail.com (J.-H.B.); raveendars@chungbuk.ac.kr (R.S.); taekyung7708@chungbuk.ac.kr (T.k.H.); 2 Department of Crop Science and Biotechnology, Dankook University, Cheonan 31116, Republic of Korea; intron@dankook.ac.kr; 3 Residual Agrochemical Assessment Division, National Institue of Agricultural Science, Rural Development Administration, Wanju 55365, Republic of Korea; peterpan7776@gmail.com; 4 Department of Information Statistics, Chungbuk National University, Cheongju 28644, Republic of Korea; 0331ost@chungbuk.ac.kr (S.O.); theo@cbnu.ac.kr (T.-Y.H.); 5 Department of Food Science and Biotechnology, Andong National University, Andong 36729, Republic of Korea; jeehye@anu.ac.kr; 6 Department of Crop Science, Chungbuk National University, Cheongju 28644, Republic of Korea; yoonsupso@chungbuk.ac.kr (Y.-S.S.); yujk0830@chungbuk.ac.kr (J.-K.Y.); 7 Department of Botany and Microbiology, College of Science, King Saud University, P.O. Box 2455, Riyadh 11451, Saudi Arabia; aalgarawi@ksu.edu.sa (A.M.A.); ahatamleh@ksu.edu.sa (A.A.H.); 8 Biomedical Institute of Mycological Resource, International St. Mary’s Hospital, College of Medicine, Catholic Kwandong University, Incheon 22711, Republic of Korea; 9 Department of Convergence Science, College of Medicine, Catholic Kwandong University, Gangneung 25601, Republic of Korea

**Keywords:** amaranth, genotype influence, polyphenol, phenolic compounds, species

## Abstract

Amaranth is a nutritionally valuable crop, as it contains phenolic acids and flavonoids, yielding diverse plant secondary metabolites (PSMs) like phytosterol, tocopherols, and carotenoids. This study explored the variations in the contents of seventeen polyphenolic compounds within the leaves of one hundred twenty *Amaranthus* accessions representing nine *Amaranthus* species. The investigation entailed the analysis of phenolic content across nine *Amaranthus* species, specifically *A. hypochondriacus*, *A. cruentus*, *A. caudatus*, *A. tricolor*, *A. dubius*, *A. blitum*, *A. crispus*, *A. hybridus*, and *A. viridis*, utilizing ultra performance liquid chromatography with photodiode array detection (UPLC-PDA). The results revealed significant differences in polyphenolic compounds among accessions in which rutin content was predominant in all *Amaranthus* species in both 2018 and 2019. Among the nine *Amaranthus* species, the rutin content ranged from 95.72 ± 199.17 μg g^−1^ (*A. dubius*) to 1485.09 ± 679.51 μg g^−1^ (*A. viridis*) in 2018 and from 821.59 ± 709.95 μg g^−1^ (*A. tricolor*) to 3166.52 ± 1317.38 μg g^−1^ (*A. hypochondriacus*) in 2019. Correlation analysis revealed, significant positive correlations between rutin and kaempferol-3-O-β-rutinoside (r = 0.93), benzoic acid and ferulic acid (r = 0.76), and benzoic acid and kaempferol-3-O-β-rutinoside (r = 0.76), whereas gallic acid showed consistently negative correlations with each of the 16 phenolic compounds. Wide variations were identified among accessions and between plants grown in the two years. The nine species and one hundred twenty *Amaranthus* accessions were clustered into six groups based on their seventeen phenolic compounds in each year. These findings contribute to expanding our understanding of the phytochemical traits of accessions within nine *Amaranthus* species, which serve as valuable resources for *Amaranthus* component breeding and functional material development.

## 1. Introduction

Polyphenols or phenolic compounds are present throughout the plant as secondary metabolites that play important roles in the plant’s defense mechanisms against stress, pathogens, and UV radiation [1]. In food, polyphenols contribute to taste, color, and stability and various studies suggest that diets high in plant polyphenols offer protection against numerous diseases [2]. More than 10,000 types of polyphenolic compounds have been identified so far, and they are classified into four types according to their structural characteristics: phenolic acids, flavonoids, stilbenes, and lignans. The determination of polyphenol content in diverse plant species with significant utility is facilitated by studies employing analytical instruments like spectrophotometers, liquid chromatography (LC), and gas chromatography (GC). These investigations, conducted across a spectrum of grain, vegetable, and fruit crops, aim to characterize the specific types and concentrations of polyphenols present in each plant, providing valuable insights into their compositional variations [3,4,5,6].

Amaranth has attracted worldwide attention due to its identification as a source of diverse secondary metabolites, encompassing phytosterols, tocopherols, carotenoids, phenolic acids, and flavonoids [7]. Amaranth, a C_4_ plant classified within the *Amaranthus* genus, encompasses around 70 species. Although its primary cultivation occurs in Central and South America, its notable adaptability enables successful growth in diverse environments, extending to temperate–tropical regions globally [8,9]. Three prominent grain *Amaranthus* species extensively cultivated are *A. caudatus*, *A. cruentus*, and *A. hypochondriacus*. In addition to these, 17 other species are specifically cultivated for their leaves [10]. During the flowering stage, the leaves of amaranth are abundant in vitamins, minerals, and dietary fiber [11]. They exhibit notable moisture and protein content, rendering them well-suited for various applications such as salads, green vegetables, animal feed, and other culinary uses [12,13]. Additionally, amaranth leaves have been found to possess pharmacological values, such as cholesterol reduction, anticancer, and anti-inflammatory properties [14], which can be demonstrated through the high polyphenol content in amaranth [3].

In *Amaranthus* species, both leaves and flowers are characterized by a high content of flavonoids, with rutin emerging as the predominant compound [15,16]. Analyzing the polyphenol content of *Amaranthus* plants across various growth stages reveals distinct patterns. Phenolic acids, specifically feruloylquinic acid and hydroxycinnamic acid, are notably high during the vegetative growth phase. In contrast, flavonoids such as rutin and quercetin become abundant during the flowering stage [17]. Furthermore, it has been observed that the polyphenol content in amaranth leaves is subject to variations influenced by both biotic and abiotic stresses, even when derived from the same resource throughout the growth period [18,19,20]. Hence, recognizing the significant differences in polyphenol content among accessions, evaluating different species of *Amaranthus* is crucial to identify potential variations among them.

The characteristics and concentrations of polyphenols in amaranth leaves can vary depending on the growth environment, necessitating a comparative analysis of findings from different studies. Previous research has focused on quantifying polyphenol content in specific amaranth species and resources [17]. However, discrepancies in sampling procedures, analysis equipment, and methodologies across studies pose challenges in evaluating and comparing new resources based on prior results [21]. This study aims to identify polyphenolic compounds in accessions from nine Amaranthus species. Additionally, we analyze data variations from plants grown in two different years and assess correlations between polyphenolic compounds. The outcomes of this research will contribute valuable insights to breeding initiatives targeting the development of amaranth accessions with enhanced nutritive value.

## 2. Materials and Methods

### 2.1. Plant Materials and Growth Conditions

**Figure 1:**
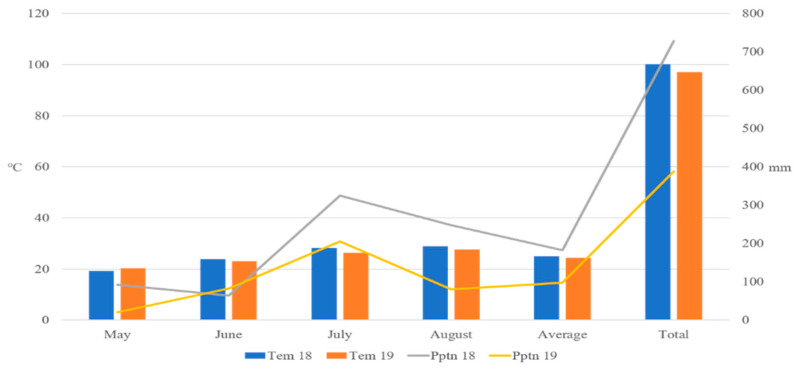
Temperature and precipitation data in Cheongju over two years (2018 and 2019). Total represents the sum of temperature and precipitation during the Amaranthus growing season (May to August). Tem: temperature; Pptn: precipitation.

A total of 120 accessions from 9 *Amaranthus* species were obtained from the National Agrobiodiversity Center (NAS; http://genebank.rda.go.kr; accessed on 10 January 2022) of the Rural Development Administration (RDA) in the Republic of Korea (Supplementary Table S1). The n9 *Amaranthus* species included 3 *A. blitum*, 18 *A. caudatus*, 11 *A. crispus*, 7 *A. cruentus*, 6 *A. dubius*, 7 *A. hybridus*, 31 *A. hypochondriacus*, 30 *A. tricolor,* and 7 *A. viridis* accessions. Two weeks after germination, these accessions were transplanted and cultivated in silt loam soil at the experimental field of Chungbuk National University in Korea (36°37′27.7″ N 127°27′15.3″ E) in 2018 and 2019. To enhance the integrity of each accession, six individual plants were established through the transplantation of individual *Amaranthus* seedlings at 20 cm intervals within the furrows of designated rows, with weekly irrigation applied. Fertilizer was not applied during the experiment to determine the actual genotypic differences throughout the harvesting year. Temperature and precipitation data recorded in Cheongju during the amaranth flowering period (May to August) in 2018 and 2019 were obtained from the Korea Meteorological Administration (Figure 1).

### 2.2. Sampling and Content Extraction

The sampling was conducted three months after planting, wherein the leaves of six plants per accession were freeze-dried using a FreeZone Freeze Dry System (Labconco, Kansas City, MO, USA), ground into powder, and homogenized; only this conjugated sample was gathered for UPLC-PDA analysis. To extract polyphenols, 100 mg of powdered leaf tissue was combined with 1 mL of 75% methanol and subjected to one hour of sonication. Afterward, the mixture underwent centrifugation at 12,000 rpm for 10 min. The resulting clear supernatant was filtered through a 0.2 μm filter into a clean tube, serving as the sample for determining the polyphenol content.

### 2.3. Sample Analysis by UHPLC-PDA

An ultra performance liquid chromatography (UPLC) system (Waters, Milford, MA, USA) equipped with a binary solvent delivery pump, auto-sampler, and a photodiode array (PDA) detector was used to identify individual polyphenols, as described previously [22]. The UPLC settings used in this study are summarized in Table 1. The mobile phase consisted of a binary solvent system comprising water (Solvent A) and acetonitrile (Solvent B) supplemented with 0.1% formic acid. A total of 17 phenolic compounds such as, gallic acid, 3,4-dihydroxybenzoic acid, 4-hydroxybenzoic acid, 2,4-dihydroxybenzoic acid, vanillic acid, caffeic acid, syringic acid, p-coumaric acid, ferulic acid, sinapic acid, rutin, quercetin 3-β-D-glucoside, benzoic acid, kaempferol 3-O-β-rutinoside, quercetin, cinnamic acid, and kaempferol were purchased from Sigma-Aldrich (St. Louis, MO, USA) and used as standards. For each standard compound, a methanol solution was prepared, resulting in a final concentration of 1000 ng/mL. A mixture of all standard compounds was then utilized to establish the calibration curve. The polyphenol content of each sample was calculated using the method described previously [22] with minor modification.

### 2.4. Statistical Analysis

To enable comparisons across various phenolic compounds, an analysis of variance (ANOVA) for significance *p* < 0.05 and Duncan’s multiple range test were carried out. Additionally, for correlation analysis among these compounds, PAST3 software v4.03 [23] was used for principal component analyses (PCA) and hierarchical clustering was performed using R statistical software (Version 4.2.1). The data presented in the figures and tables are represented as mean ± standard deviation. The Relative Polyphenol Content Index (RPCI) was used to compare the levels of 17 polyphenols in the samples using the following formula:

Standard score = (Absorbance − mean)/standard deviation RPCI = Average of standard score.(1)

Furthermore, a comprehensive assessment was conducted to ascertain the statistical significance of the influence exerted by the genotype, year, and their interaction (genotype × year) on the levels of 17 polyphenolic compounds.

## 3. Results

### 3.1. Polyphenolics Assessment Using the UPLC-PDA

**Figure 2:**
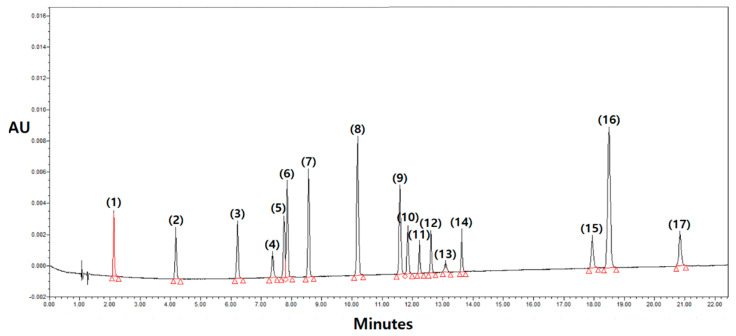
Chromatograms show the absorbance of a standard mixture of 17 polyphenols at 260 nm. The peaks were identified as follows: (1) gallic acid, (2) 3,4-dihydroxybenzoic acid, (3) 4-hydroxybenzoic acid, (4) 2,4-dihydroxybenzoic acid, (5) vanillic acid, (6) caffeic acid, (7) syringic acid, (8) p-coumaric acid, (9) feulic acid, (10) sinapic acid, (11) rutin, (12) quercetin 3-β-D-glucoside, (13) benzoic acid, (14) kaempferol 3-O-β-rutinoside, (15) quercetin, (16) cinnamic acid, and (17) kaempferol.

The compounds investigated in the methanolic extracts were quantified by integrating the peak areas at 260 nm using an external calibration method. Calibration curves were constructed individually for each standard compound to establish a relationship between concentration and peak area (Figure 2). The results obtained from the UPLC-PDA analysis, as presented in Supplementary Table S2, reveal discrepancies in polyphenol profiles across the nine cultivated *Amaranthus* species during the years 2018 and 2019, as summarized in Table 2. A comprehensive set of seventeen polyphenolic compounds was identified, encompassing seven hydroxybenzoic acids (HBA1–HBA7), five hydroxycinnamic acids (HCA1–HCA5), and five flavonoids (FLA1–FLA5). In addition, UPLC-PDA was used for the qualification and quantification of phenolic compounds in accordance with the previous method [22]. Specifically, hydroxybenzoic acids include gallic acid (HBA1), 3,4-dihydroxybenzoic acid (HBA2), 4-hydroxybenzoic acid (HBA3), 2,4-dihydroxybenzic acid (HBA4), vanillic acid (HBA5), syringic acid (HBA6), and benzoic acid (HBA7). The hydroxycinnamic acids consist of caffeic acid (HCA1), p-coumaric acid (HCA2), ferulic acid (HCA3), sinapic acid (HCA4), and cinnamic acid (HCA5). Lastly, the flavonoids identified are rutin (FLA1), quercetin-3-β-D-glucoside (FLA2), kaempferol-3-O-β-rutinoside (FLA3), quercetin (FLA4), and kaempferol (FLA5). These compounds were documented from the leaves of the nine Amaranthus species under investigation.

#### 3.1.1. Hydroxybenzoic Acid

In the analysis conducted across one hundred twenty *Amaranthus* accessions spanning the years 2018 and 2019, examination of seven hydroxybenzoic acids revealed notable variations. Gallic acid (HBA1) content was observed to be at its lowest in 2018, ranging from 1.8 to 4.5 μg g^−1^ with an average of 2.0 μg g^−1^, while 3,4-dihydroxybenzoic acid (HBA2) content was observed to be at its lowest in 2019, ranged from 2.9 to 14.8 μg g^−1^ with an average of 6.5 μg g^−1^. Conversely, benzoic acid (HBA7) exhibited the highest content across both years, with concentrations of 142.5 μg g^−1^ in 2018 and 173.5 μg g^−1^ in 2019, yielding an average of 93.6 μg g^−1^ across the assessed *Amaranthus* species (Table 2). Notably, the content of 3,4-dihydroxybenzoic acid (HBA2) remained undetectable in *A. blitum* accessions throughout the cultivation periods under scrutiny.

#### 3.1.2. Hydroxycinnamic Acid

In the comprehensive analysis conducted across one hundred twenty *Amaranthus* accessions spanning the years 2018 and 2019, scrutiny of five hydroxycinnamic acids showcased significant variability. Cinnamic acid (HCA5) content displayed its nadir ranging from 2.0 to 3.4 μg g^−1^ in 2018, averaging 1.6 μg g^−1^, and fluctuating between 1.8 to 6.3 μg g^−1^ in 2019, averaging 3.1 μg g^−1^. Conversely, sinapic acid (HCA4) consistently exhibited the highest content across both years, registering concentrations of 55.4 μg g^−1^ in 2018 and 47.8 μg g^−1^ in 2019, resulting in an average of 24.3 μg g^−1^ across the evaluated *Amaranthus* species (Table 2).

#### 3.1.3. Flavonoid

The UPLC-PDA chromatogram revealed the presence of five flavonoid compounds, which showed significant variability during the assessment period. Kaempferol (FLA5) content ranged from 2.2 to 9.3 μg g^−1^ in 2018 with an average of 3.5 μg g^−1^ and from 12.9 to 16.6 μg g^−1^ in 2019 with an average of 14.7 μg g^−1^. Remarkably, rutin (FLA1) consistently exhibited the highest content across both years and ranged from 95.7 to 1485.1 μg g^−1^ in 2018 with an average of 910.4 μg g^−1^ and from 821.6 to 3166.5 μg g^−1^ in 2019 with an average of 1869.9 μg g^−1^.

### 3.2. Relative Polyphenol Content Index

**Figure 3:**
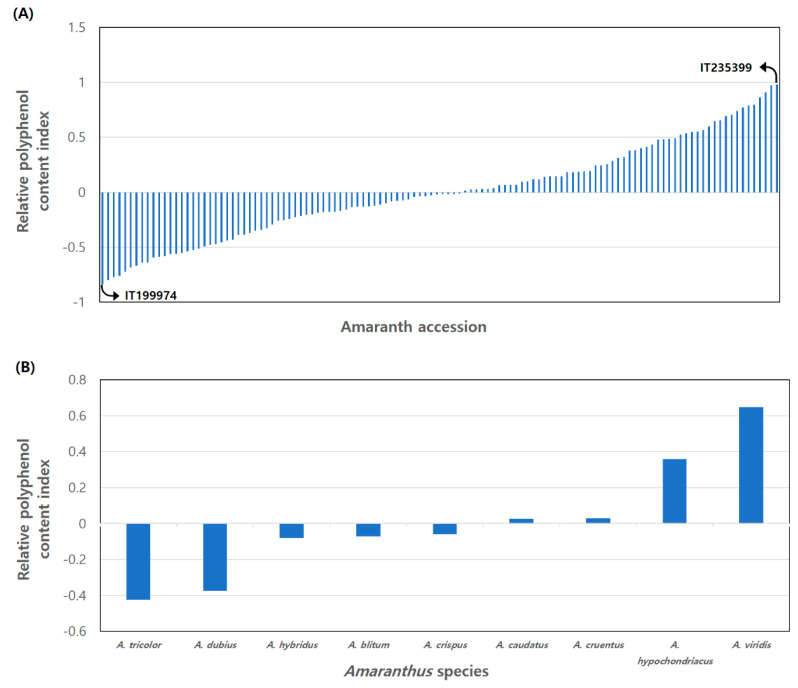
Relative Polyphenol Content Index (RPCI) of 9 *Amaranthus* species, based on the contents of 17 polyphenols in 2018 and 2019. (**A**) Comparison between 120 accessions. (**B**) Comparison between 9 species.

In 2018, *Amaranthus* accession A118 (RPCI: 2.236) exhibited the highest RPCI, followed by A120 (RPCI: 2.135), A119 (RPCI: 1.998), and A14 (RPCI: 1.667), while A56 (RPCI: −0.843) displayed the lowest RPCI. In 2019, A7 (RPCI: 1.842) demonstrated the highest RPCI, trailed by A1 (RPCI: 1.557), A12 (RPCI: 1.479), and A6 (RPCI: 1.395), whereas A81 (RPCI: −0.976) exhibited the lowest RPCI (Supplementary Table S3). The cumulative RPCI over both years was greatest for accession A119 (RPCI: 1.006), followed by A118 (RPCI: 0.975), A1 (RPCI: 0.913), and A19 (RPCI: 0.911), while A60 (RPCI: −0.833) recorded the lowest (Figure 3A). Among the nine species, *A. viridis* (RPCI: 0.621) showcased the highest RPCI, followed by *A. hypochondriacus* (RPCI: 0.456), *A. cruentus* (RPCI: 0.024), and *A. caudatus* (RPCI: 0.021), whereas *A. tricolor* (RPCI: −0.448) exhibited the lowest RPCI (Figure 3B).

### 3.3. Correlation Analysis

**Figure 4:**
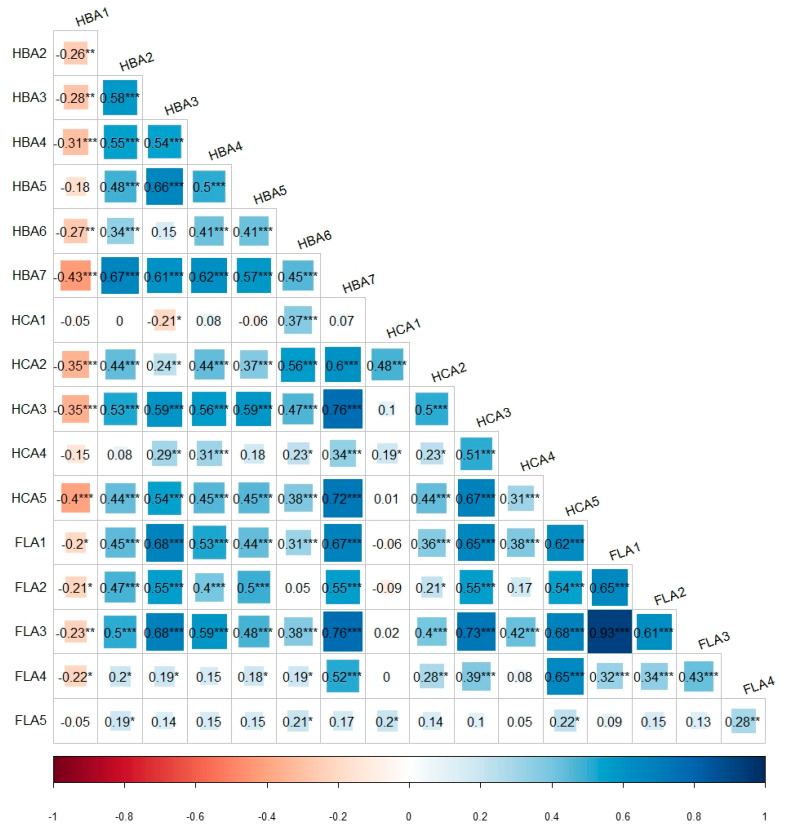
Correlations between 17 polyphenols in 9 *Amaranthus* species combining 2018 and 2019 data. HBA1: gallic acid; HBA2: 3,4-dihydroxybenzoic acid; HBA3: 4-hydroxybenzoic acid; HBA4: 2,4-dihydroxybenzic acid; HBA5: vanillic acid; HBA6: syringic acid; HBA7: benzoic acid; HCA1: caffeic acid; HCA2: p-coumaric acid; HCA3: ferulic acid; HCA4: sinapic acid; HCA5: cinnamic acid; FLA1: rutin; FLA2: quercetin-3-β-D-glucoside; FLA3: kaempferol-3-O-β-rutinoside; FLA4: quercetin; FLA5: kaempferol. *: *p* < 0.01, **: *p* < 0.05, and ***: *p* < 0.001.

The correlation analysis (Figure 4) of polyphenol content data over two years unveiled significant positive correlations (*p* < 0.001) between rutin and kaempferol-3-O-β-rutinoside (r = 0.93), benzoic acid and ferulic acid (r = 0.76), as well as benzoic acid and kaempferol-3-O-β-rutinoside (r = 0.76). However, gallic acid displayed notably negative correlations (*p* < 0.001) with benzoic acid (r = −0.43), cinnamic acid (r = −0.40), p-coumaric acid (r = −0.35), and ferulic acid (r = −0.35).

### 3.4. Principal Component Analysis

**Figure 5:**
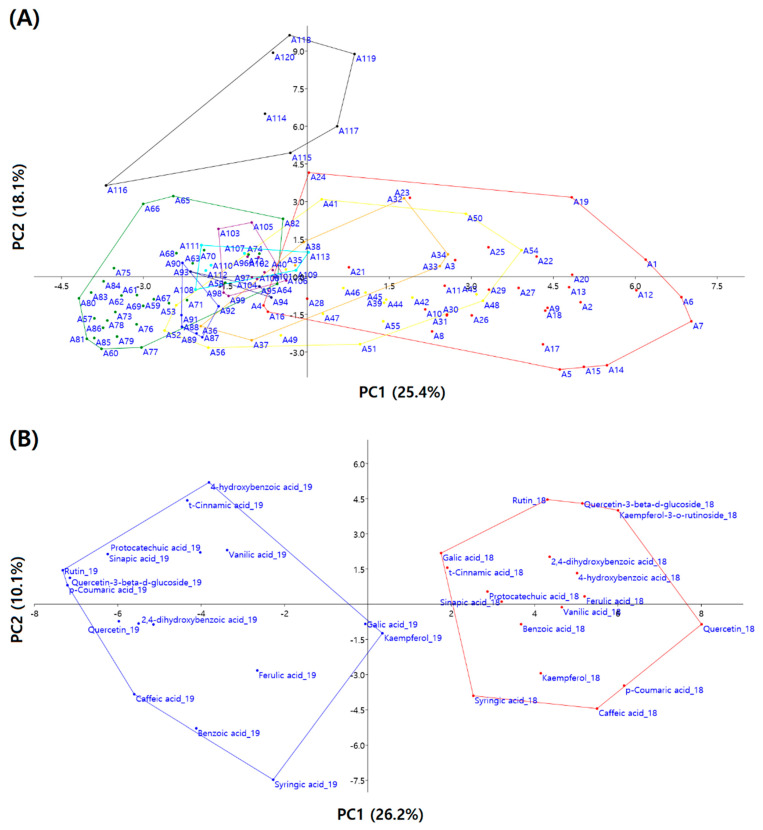
Principal component analysis (PCA) of ni9 *Amaranthus* species, based on the contents of 17 polyphenols in 2018 and 2019. (**A**) Comparison between 120 accessions. Red, *A. hypochondriacus*; Orange, *A. cruentus*; Yellow, *A. caudatus*; Green, *A. tricolor*; Blue, *A. dubius*; Navy, *A. blitum*; Purple, *A. crispus*; Aqua, *A. hybridus*; Black, *A. viridis*. (**B**) Comparison between two years. Red, 2018; Blue, 2019.

PCA was conducted to compare the contents of seventeen polyphenols among one hundred twenty *Amaranthus* accessions. The analysis revealed eight principal components (PCs), each with eigenvalues > 1.0, collectively explaining 71.69% of the total variance in polyphenol content (Table 3). PC1 (eigenvalue = 7.98) accounted for 25.44% of the total variance. Benzoic acid_19 (0.326) exhibited the highest positive variance, while quercetin_18 (−0.149) displayed the highest negative variance. PC2 (Eigenvalue = 5.67) explained an additional 18.13% of the total variance. Kaempferol-3-O-β-rutinoside_18 (0.323) demonstrated the highest positive variance, whereas cinnamic acid_19 (−0.107) exhibited the highest negative variance. PC3 (Eigenvalue = 2.42) explained an additional 7.71% of the total variance. Caffeic acid_19 (0.421) showed the highest positive variance, while 4-hydroxybenzoic acid_19 (−0.261) displayed the highest negative variance. Moreover, the PCA results unveiled annual variation and diverse patterns that were not segregated by species (Figure 5).

### 3.5. Heatmap Hierarchical Clustering

**Figure 6:**
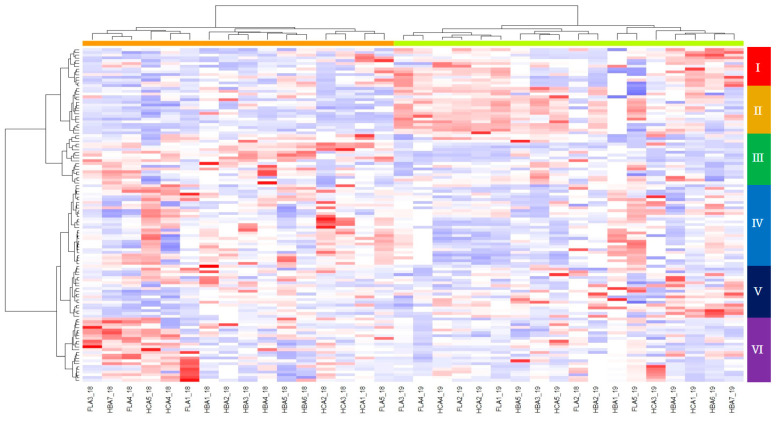
Hierarchical clustering analysis of 9 *Amaranthus* species, based on the contents of 17 polyphenol contents in 2018 and 2019. HBA1: gallic acid; HBA2: 3,4-dihydroxybenzoic acid; HBA3: 4-hydroxybenzoic acid; HBA4: 2,4-dihydroxybenzic acid; HBA5: vanillic acid; HBA6: syringic acid; HBA7: benzoic acid; HCA1: caffeic acid; HCA2: p-coumaric acid; HCA3: ferulic acid; HCA4: sinapic acid; HCA5: cinnamic acid; FLA1: rutin; FLA2: quercetin-3-β-D-glucoside; FLA3: kaempferol-3-O-β-rutinoside; FLA4: quercetin; FLA5: kaempferol.

The one hundred twenty *Amaranthus* accessions were categorized into six groups based on their contents of seventeen polyphenols (Table 4 and Figure 6). Group I comprised 14 accessions characterized by high contents of syringic acid (15.7 ± 11.6 μg g^−1^), caffeic acid (16.4 ± 15.0 μg g^−1^), and p-coumaric acid (16.8 ± 12.1 μg g^−1^). Group II, consisting of 17 accessions, showed low contents of gallic acid (1.2 ± 1.9 μg g^−1^) and caffeic acid (8.1 ± 6.8 μg g^−1^) but high contents of 3,4-dihydroxybenzoic acid (11.4 ± 7.2 μg g^−1^), 3,4-hydroxybenzoic acid (64.2 ± 58.8 μg g^−1^), benzoic acid (133.9 ± 84.2 μg g^−1^), ferulic acid (32.3 ± 21.5 μg g^−1^), cinnamic acid (4.5 ± 3.8 μg g^−1^), rutin (2115.2 ± 1579.9 μg g^−1^), quercetin-3-β-D-glucoside (221.0 ± 174.1 μg g^−1^), kaempferol-3-O-β-rutinoside (112.6 ± 77.8 μg g^−1^), and quercetin (41.6 ± 34.5 μg g^−1^). Group III, with 18 accessions, demonstrated the highest contents of 2,4-dihydroxybenzic acid (37.8 ± 28.3 μg g^−1^), vanillic acid (40.3 ± 33.4 μg g^−1^), and kaempferol (10.7 ± 8.0 μg g^−1^). Group IV (17 accessions) had the lowest contents of 3,4-dihydroxybenzoic acid (1.8 ± 2.0 μg g^−1^), 4-hydroxybenzoic acid (7.3 ± 4.9 μg g^−1^), 2,4-dihydroxybenzic acid (7.5 ± 6.6 μg g^−1^), vanillic acid (9.8 ± 7.4 μg g^−1^), syringic acid (3.3 ± 2.1 μg g^−1^), benzoic acid (34.4 ± 23.0 μg g^−1^), p-coumaric acid (5.6 ± 4.1 μg g^−1^), ferulic acid (12.3 ± 8.7 μg g^−1^), sinapic acid (10.4 ± 15.7 μg g^−1^), cinnamic acid (0.9 ± 1.2 μg g^−1^), rutin (553.5 ± 535.4 μg g^−1^), quercetin-3-β-D-glucoside (43.6 ± 49.5 μg g^−1^), and kaempferol-3-O-β-rutinoside (41.9 ± 23.9 μg g^−1^) but had the highest content of gallic acid (4.2 ± 3.7 μg g^−1^). Group V (19 accessions) showed the lowest contents of quercetin (17.4 ± 11.1 μg g^−1^) and kaempferol (7.0 ± 6.5 μg g^−1^). Group VI (23 accessions) showed the highest content of sinapic acid (32.8 ± 30.5 μg g^−1^).

### 3.6. Statistical Analysis of Genotype, Year, and Genotype × Year across 17 Polyphenols

In this study, we conducted an examination of the statistical significance of genotype, year, and genotype × year variables for 17 types of polyphenols. The findings revealed very high statistical significance for all variables across all polyphenols (Table 5). However, sinapic acid and kaempferol exhibited differences from the other 15 polyphenols, demonstrating comparatively lower significance in both year and genotype, respectively. These results suggest that sinapic acid and kaempferol may possess unique characteristics compared to the rest of the polyphenols. The study underscores the potential variation in the impact of genotype and year based on the type of polyphenol, indicating the need for further investigation into the distinctive properties and roles of sinapic acid and kaempferol in the context of polyphenol metabolism and biological activity.

## 4. Discussion

The study found that all genotypes have similar polyphenol profiles, but there were significant differences in polyphenol concentrations between years and genotypes. During the flowering period from the month of May to August, total precipitation decreased by approximately 50% in 2019 (388.1 mm) when compared to 2018 (728.1 mm), while the total temperature remained relatively stable, with no significant change between 2018 and 2019 with an average of 25 and 24.2 °C, respectively (Figure 1). Sarker and Oba, (2018) reported increased antioxidant activities and 16 polyphenol contents with an increase in drought stress [20], and Barba de la Rosa et al. (2019) mentioned that, in addition to drought stress, external factors like insect damage, light limitation, and nutrient limitation can alter the polyphenol content of a given accession [18].

Numerous scientific reports have addressed the influence of factors such as total phenolic and anthocyanin content, maturity, and diverse plant species on antioxidant capacity [10,24]. Phenolic compounds emerge as the predominant antioxidant components, contributing to robust antioxidant activity and stress response in various tested plants [25]. To harness the potential of these substantial natural antioxidant sources, additional characterization of the phenolic composition is essential [3]. In this study, we observed a broad variation in the content of seven hydroxybenzoic acids, five hydroxycinnamic acids, and five flavonoids across one hundred twenty accessions representing nine different *Amaranthus* species.

Plants accumulate phenolic compounds in response to various stress and climatic conditions, leading to increased production of reactive oxygen and nitrogen species within the host plant’s body [26]. Due to the variations in type and content among different plant species, studies measuring content are being conducted on various plant species to comprehensively understand and quantify these differences [27,28,29]. Previous studies have identified similarities between quinoa and *Amaranthus* species in terms of several phenolic acids, flavonoids, and their glycosides [7,30]. Similarly, in a study by Khanam et al. (2012), higher contents of gallic acid, vanillic acid, 4-hydroxybenzoic acid, caffeic acid, ferulic acid, sinapic acid, cinnamic acid, and quercetin-3-glucoside were reported in *Amaranthus* leaves compared to other leafy greens, including komatsuna, mizuna, pok choi, mitsuba, salad spinach, and lettuce [3]. This emphasizes the substantial polyphenol content of *Amaranthus* and its potential applications.

The levels of various polyphenols in *A. hypochondriacus* and *A. tricolor* in this study were found to be higher than those reported by previous studies [3,18,31]. Furthermore, in comparison with previous studies, the levels of 14 types of polyphenols identified in this study were 2–5 times higher, with rutin’s level exceeding 10 times the previously reported amounts. This study facilitates a comprehensive and simultaneous comparison of polyphenol contents among various amaranth species and accessions, offering crucial insights for material development.

This study’s findings also confirmed notably high rutin content in *Amaranthus* compared to other polyphenols. Recognizing the substantial differences in polyphenol contents among accessions, adjustments were essential to mitigate potential impacts on the overall analysis. Similar to the antioxidant assay utilizing the Relative Antioxidant Capacity Index (RACI) to account for systematic differences in various antioxidant experiments [32], we introduced the concept of the Relative Polyphenol Content Index (RPCI). The highest RPCI values were observed in *A. viridis* (1.65) in 2018 and *A. hypochondriacus* (0.98) in 2019. The overall RPCI was elevated in *A. viridis* and three grain *Amaranthus* species. Despite a decrease in *A. viridis’* polyphenol content in 2019 compared to 2018, its total RPCI value remained the highest among the nine species, indicating that its polyphenol content surpassed that of other species in both years. Seasonal variation significantly influencing flavonoid biosynthesis in *Tetrastigma hemsleyanum* Diels & Gilg was reported [33].

In this study, a notable and statistically robust positive correlation was observed between rutin and kaempferol-3-O-β-rutinoside, with a correlation coefficient (r) of 0.93. Rutin and kaempferol-3-O-β-rutinoside are rutinosides of quercetin and kaempferol, respectively, which have independent pathways but go through the same biosynthetic mechanism [34]. Interestingly, a strong positive correlation (r = 0.98) of rutin and kaempferol-3-O-β-rutinoside content was observed in *A. hybridus* and *A. caudatus* in the present study. Furthermore, a previous study by Chen et al. (2018) reported high accumulations of rutin and kaempferol 3-O-rutinoside in the Wuyi Rock tea cultivar, grown in the same environmental conditions subjected to the same cultivation practices, further supporting the observed correlation in our study [35]. However, it is noteworthy that Gallic acid and benzoic acid displayed the strongest negative correlation (r = −0.43) in our study. Gallic acid also exhibited negative correlations with other polyphenols, likely due to its less frequent detection in 2019 compared to 2018 (Supplementary Table S4). Numerous investigations have explored the impact of seasonal variations on the production of plant secondary metabolites, specifically focusing on the accumulation of specific compounds in plants [36,37,38].

Hierarchical clustering analysis and PCA confirmed annual variation, dividing the accessions into six groups, independent of their species (Figure 4). Grain *Amaranthus* accessions were evenly distributed in groups 1, 2, 3, and 5, while all *A. viridis* and *A. biltum* accessions were clustered in groups 3 and 6, respectively. Groups 4 and 6 included only vegetable *Amaranthus* accessions. Group 5 was the most diverse, containing accessions belonging to six of the nine species. Compared with 2018, the polyphenol contents of groups 3, 4, and 6 were lower in 2019, while those of groups 1, 2, and 5 were higher in 2019. In contrast, flavonoid contents were higher in 2019 than in 2018 for all groups (Table 4). The inheritability of chemical traits in plants generally surpasses that of morphological, phenological, and life-history traits [39]. This high heritability in chemical traits likely indicates high evolvability, despite potential sensitivity to environmental variation in heritability measures [40]. Studies consistently demonstrate high or moderate estimates of heritability for PSMs production across various plant tissues, including leaves [41]. The significant variability between the year and genotypes variables for 17 types of polyphenols may contribute to these high heritabilities, suggesting ample genetic variation between species. Similarly, in our study, clustering and PCA analysis confirmed variation, dividing the accessions into six groups independent of their species.

## 5. Conclusions

The findings of this study underscore the potential of one hundred twenty *Amaranthus* accessions spanning nine different species as valuable sources of polyphenols. Despite the high protein and nutrient contents of various *Amaranthus* species, the genus remains underutilized and often overlooked. Considering that the Amaranthaceae family comprises approximately 70 *Amaranthus* species, with 20 producing edible leaves and/or grains [31], our study provides a comprehensive evaluation of polyphenol content across different *Amaranthus* species under varying environmental conditions. These findings unveil new potential applications of *Amaranthus* spp. For instance, our study highlights that *A. viridis* and *A. hypochondriacus* exhibited higher polyphenol content in 2018 and 2019, respectively, compared to other Amaranthus species. Similarly, based on the overall harvest years (2018–2019) among the nine species, *A. viridis* showcased the highest RPCI, followed by *A. hypochondriacus*, suggesting their potential for the development of new health-promoting materials. Overall, the results of this study provide essential insights that can guide decision-making processes in *Amaranthus* breeding programs.

## Figures and Tables

**Table 1:** UPLC-PDA operating conditions.

Parameters	Conditions	
UPLC-PDA conditions below		
Injection volume	5 μL	
Column temperature	40 °C	
Flow rate	0.25 mL/min	
Column type	BEH C18 column (2.1 mm × 100 mm, 1.7 μm)	
**Gradient/mobile phase Time (min)**	**Solvent A (%)**	**Solvent B (%)**
0	98	2
20	75	25
24	40	60
27	10	90
28	10	90
30	98	2
35	re-equilibration	

**Table 2:** Descriptive statistics of the contents of 17 polyphenols in leaf extracts of 9 *Amaranthus* species in 2018 and 2019.

Phenolic Compound	Year	Species									
		*A. hypo*	*A. crue*	*A. caud*	*A. tric*	*A. dubi*	*A. blit*	*A. cris*	*A. hybr*	*A. viri*	Average
**Hydroxybenzoic acid (μg g^−1^)**											
HBA1	2018	3.0 ± 1.5 ab ^1^	4.5 ± 3.9 a	2.8 ± 1.6 ab	2.3 ± 0.5 b	1.8 ± 0.3 b	1.9 ± 0.4 b	2.0 ± 0.5 b	4.5 ± 2.0 a	4.3 ± 0.5 a	2.0 ± 1.8 g
	2019	24.2 a	7.7 ± 8.7 b	7.5 ± 7.5 b	9.2 ± 0.5 b	8.6 b	9.0 ± 0.7 b	8.8 ± 0.3 b	9.5 ± 0.9 b	8.9 ± 0.2 b	4.3 ± 5.3 fg
HBA2	2018	6.2 ± 3.9 bc	6.1 ± 5.1 bc	5.4 ± 2.3 bc	3.0 ± 1.3 c	1.9 ± 0.4 c	-	2.5 ± 1.0 c	7.5 ± 5.2 b	16.9 ± 4.6 a	4.1 ± 4.8 fg
	2019	14.8 ± 6.0 a	9.0 ± 4.5 b	8.5 ± 6.1 b	3.5 ± 1.4 b	4.2 ± 0.9 b	-	2.9 ± 0.2 b	5.6 ± 0.3 b	3.9 ± 1.2 b	6.5 ± 6.9 fg
HBA3	2018	11.1 ± 4.8 bc	13.3 ± 4.9 b	10.1 ± 9.9 bc	6.9 ± 4.5 bc	10.2 ± 7.4 bc	5.4 ± 1.3 c	6.9 ± 3.9 bc	7.1 ± 2.6 bc	31.9 ± 11.0 a	10.3 ± 8.4 ef
	2019	108.3 ± 43.5 a	78.9 ± 33.7 ab	36.1 ± 33.6 cd	13.0 ± 12.4 d	6.3 ± 1.2 d	37.2 ± 16.7 cd	61.8 ± 18.5 bc	27.9 ± 23.5 d	38.8 ± 28.1 cd	51.1 ± 47.6 b
HBA4	2018	24.6 ± 28.4 b	18.4 ± 6.4 b	16.1 ± 11.8 b	9.2 ± 14.1 b	13.7 ± 10.1 b	13.6 ± 13.4 b	17.6 ± 7.7 b	18.1 ± 7.9 b	49.4 ± 14.2 a	18.3 ± 19.5 d
	2019	42.7 ± 15.9 a	19.8 ± 15.6 b	38.1 ± 14.2 a	10.6 ± 12.3 b	12.4 ± 8.1 b	21.8 ± 3.6 b	21.1 ± 10.2 b	23.6 ± 19.7 b	16.8 ± 10.6 b	25.7 ± 19.0 c
HBA5	2018	16.6 ± 5.6 bc	22.7 ± 5.9 b	19.4 ± 6.7 bc	13.5 ± 8.6 cd	4.1 ± 0.5 e	13.7 ± 14.9 cd	5.9 ± 3.8 de	19.8 ± 8.9 bc	46.5 ± 14.3 a	16.8 ± 11.5 de
	2019	52.2 ± 12.9 a	37.4 ± 24.0 ab	40.1 ± 41 ab	9.4 ± 5.5 cd	6.5 ± 1.9 d	19.0 ± 3.4 bcd	33.0 ± 26.1 abc	18.3 ± 15.4 bcd	36.7 ± 31.9 ab	30.9 ± 27.2 c
HBA6	2018	4.9 ± 4.3 b	6.0 ± 5.9 b	6.0 ± 3.5 b	2.9 ± 0.9 b	2.7 ± 1.2 b	4.5 ± 1.4 b	4.6 ± 1.6 b	4.5 ± 1.4 b	10.6 ± 5.7 a	4.5 ± 3.8 fg
	2019	13.4 ± 10.6 ab	6.2 ± 1.6 bc	20.2 ± 6.8 a	5.0 ± 3.4 bc	6.3 ± 2.2 bc	6.8 ± 4.0 bc	6.9 ± 3.5 bc	4.1 ± 1.0 c	5.3 ± 3.1 bc	9.5 ± 8.7 fg
HBA7	2018	61.7 ± 22.2 b	64.3 ± 25.8 b	64.2 ± 33.2 b	39.7 ± 21.7 bc	27.5 ± 4.0 c	33.3 ± 9.2 bc	39.5 ± 28.5 bc	52.3 ± 13.2 bc	142.5 ± 65.7 a	56.5 ± 36.6 b
	2019	173.5 ± 59.2 a	110.5 ± 50.3 b	109.9 ± 55.3 b	32.5 ± 24.0 c	74.2 ± 19.2 bc	57.9 ± 3.1 c	61.2 ± 16.1 c	74.8 ± 18.2 bc	44.7 ± 21.7 c	93.6 ± 68.1 a
**Hydroxycinnamic acid (μg g^−1^)**											
HCA1	2018	5.2 ± 4.4 c	5.1 ± 2.2 c	4.5 ± 4.2 c	9.5 ± 10.3 bc	18.4 ± 16.3 ab	14.4 ± 3.7 bc	13.8 ± 4.9 bc	10.8 ± 7.2 bc	27.1 ± 23.0 a	9.3 ± 10.6 cd
	2019	14.6 ± 12.8 ab	9.4 ± 7.8 b	20.9 ± 13.2 a	8.2 ± 4.6 b	12.4 ± 5.2 ab	7.9 ± 0.7 b	8.9 ± 5.2 b	9.0 ± 4.3 b	9.2 ± 3.3 b	12.1 ± 10.0 c
HCA2	2018	6.3 ± 4.3 b	7.8 ± 4.1 b	7.1 ± 5.1 b	7.8 ± 4.9 b	5.0 ± 1.3 b	7.0 ± 0.6 b	5.3 ± 1.5 b	5.2 ± 0.7 b	24.9 ± 12.7 a	7.4 ± 6.7 d
	2019	17.8 ± 8.0 a	10.6 ± 8.5 b	18.1 ± 11.2 a	4.0 ± 1.9 b	8.5 ± 1.5 b	5.6 ± 0.6 b	4.3 ± 0.8 b	6.0 ± 1.6 b	5.8 ± 2.6 b	10.5 ± 9.0 cd
HCA3	2018	17.5 ± 7.1 bc	20.3 ± 9.2 bc	17.7 ± 9.8 bc	15.6 ± 10.9 c	17.0 ± 10.5 bc	12.8 ± 10.3 c	26.1 ± 6.2 b	19.3 ± 8.5 bc	35.6 ± 10.2 a	19.0 ± 10.2 b
	2019	41.0 ± 16.9 a	25.7 ± 7.1 bc	28.3 ± 9.9 b	9.8 ± 6.8 d	18.9 ± 1.1 bcd	18.7 ± 8.6 bcd	19.9 ± 1.9 bcd	19.0 ± 5.8 bcd	15.0 ± 6.4 cd	24.0 ± 15.6 a
HCA4	2018	17.6 ± 9.6 b	21.1 ± 13.8 b	15.8 ± 11.5 b	15.8 ± 26.2 b	14.0 ± 6.8 b	51.5 ± 41.9 a	55.4 ± 29.2 a	14.4 ± 13.4 b	29.6 ± 15.6 b	20.3 ± 22.0 b
	2019	33.8 ± 14.2 ab	29.1 ± 22.6 ab	24.2 ± 14.0 b	14.5 ± 16.2 b	33.8 ± 9.5 ab	47.8 ± 22.7 a	33.0 ± 19.0 ab	21.8 ± 9.3 b	24.1 ± 30.3 b	24.3 ± 19.2 a
HCA5	2018	2.4 ± 0.6 a	2.6 ± 0.6 a	2.5 ± 0.6 a	2.3 ± 0.8 a	2.0 ± 0.7 a	2.8 ± 0.9 a	2.4 ± 0.6 a	2.4 ± 0.7 a	3.4 ± 2.6 a	1.6 ± 1.4 e
	2019	6.3 ± 2.8 a	3.9 ± 1.7 ab	4.1 ± 1.6 ab	2.6 ± 0.7 b	1.8 ± 0.2 b	2.8 ± 0.5 b	2.3 ± 0.5 b	2.3 ± 0.4 b	1.9 ± 0.3 b	3.1 ± 2.8 e
**Flavonoid (μg g^−1^)**											
FLA1	2018	1072.3 ± 799.9 ab	788.1 ± 767.4 abc	614.6 ± 485.4 bc	831.3 ± 854.1 abc	95.7 ± 199.2 c	973.3 ± 391.0 ab	1162.0 ± 495.8 ab	1116.6 ± 411.5 ab	1485.1 ± 679.5 a	910.4 ± 736.9 b
	2019	3166.5 ± 1317.4 a	2433.9 ± 576.4 ab	1875.5 ± 816.0 bc	821.6 ± 710.0 d	1360.0 ± 348.6 cd	1915.7 ± 941.4 bc	1716.7 ± 434.6 bcd	1368.6 ± 427.3 cd	1201.0 ± 589.4 cd	1869.9 ± 1234.9 a
FLA2	2018	74.4 ± 49.2 bc	97.5 ± 86.2 bc	44.7 ± 30.8 bc	48.5 ± 52.2 bc	23.2 ± 33.4 c	55.2 ± 26.4 bc	129.2 ± 160.2 b	123.8 ± 104.3 b	224.0 ± 157.2 a	78.4 ± 89.4 cd
	2019	310.1 ± 118.1 a	229.0 ± 154.8 ab	112.9 ± 56.2 c	72.6 ± 68.5 c	140.7 ± 65.7 bc	137.7 ± 51.2 bc	181.4 ± 111.3 bc	177.3 ± 95.8 bc	182.2 ± 117.6 bc	176.6 ± 130.4 c
FLA3	2018	54.2 ± 33.2 bc	47.3 ± 30.0 bc	35.3 ± 21.6 c	51.5 ± 33.1 bc	34.4 ± 15.5 c	57.5 ± 16.3 bc	71.0 ± 27.6 ab	57.6 ± 19.7 bc	100.0 ± 41.2 a	53.8 ± 32.7 cd
	2019	161.1 ± 61.7 a	112.0 ± 35.1 b	109.3 ± 43.4 b	46.9 ± 26.9 c	71.6 ± 10.6 bc	105.0 ± 45.4 b	84.1 ± 14.8 bc	68.8 ± 17.7 bc	57.6 ± 26.9 c	97.6 ± 59.2 cd
FLA4	2018	9.3 ± 6.0 c	10.5 ± 10.2 c	13.9 ± 8.5 bc	23.7 ± 10.7 a	22.7 ± 1.5 ab	28.0 ± 0.7 a	24.1 ± 4.2 a	22.5 ± 3.9 ab	31.5 ± 19.1 a	18.1 ± 11.3 d
	2019	56.2 ± 28.1 a	23.2 ± 19.7 b	41.4 ± 24.1 ab	28.8 ± 4.2 b	30.6 ± 2.3 b	32.0 ± 0.7 b	30.7 ± 2.3 b	32.4 ± 3.4 b	29.7 ± 3.0 b	38.1 ± 21.1 d
FLA5	2018	3.7 ± 3.3 b	2.2 ± 0.7 b	4.2 ± 2.6 b	3.6 ± 1.3 b	2.9 ± 0.4 b	3.0 ± 0.3 b	2.8 ± 0.1 b	3.3 ± 1.4 b	9.3 ± 5.0 a	3.5 ± 2.8 d
	2019	16.2 ± 10.6 a ^1^	12.9 ± 7.1 a	13.1 ± 7.7 a	14.3 ± 3.0 a	16.0 ± 0.6 a	16.6 ± 0.4 a	16.5 ± 0.5 a	13.8 ± 3.4 a	13.8 ± 3.3 a	14.7 ± 6.8 d
Average	2018	81.82 ± 58.15 a	66.93 ± 61.35 a	52.02 ± 38.18 a	63.95 ± 62.12 a	17.48 ± 19.31 a	79.87 ± 33.29 a	92.42 ± 48.34 a	87.63 ± 37.75 a	133.68 ± 63.65 ab	
	2019	250.16 ± 108.67 b	185.84 ± 57.60 b	147.54 ± 68.34 b	65.09 ± 53.08 a	106.64 ± 29.93 b	152.59 ± 68.98 ab	134.91 ± 39.16 ab	110.75 ± 38.12 ab	99.73 ± 51.72 a	

^1^ The same letter in each column indicates no significant difference per Duncan’s multiple range test, *p* < 0.05. *A. hypo*: *A. hypochondriacus*; *A. crue*: *A. cruentus*; *A. caud*: *A. caudatus*; *A. tric*: *A. tricolor*; *A. dubi*: *A. dubius*; *A. blit*: *A. blitum*; *A. cris*: *A. crispus*; *A. hybr*: *A. hybridus*; *A. viri*: *A. viridis*; HBA1: gallic acid; HBA2: 3,4-dihydroxybenzoic acid; HBA3: 4-hydroxybenzoic acid; HBA4: 2,4-dihydroxybenzic acid; HBA5: vanillic acid; HBA6: syringic acid; HBA7: benzoic acid; HCA1: caffeic acid; HCA2: p-coumaric acid; HCA3: ferulic acid; HCA4: sinapic acid; HCA5: cinnamic acid; FLA1: rutin; FLA2: quercetin-3-β-D-glucoside; FLA3: kaempferol-3-O-β-rutinoside; FLA4: quercetin; FLA5: kaempferol.

**Table 3:** Eigenvalue and component matrix of the principal component (PC) axes and total variation explained by each PC.

Principal Components	PC1	PC2	PC3	PC4	PC5	PC6	PC7	PC8
Eigen value	7.98	5.68	2.42	1.66	1.37	1.27	1.09	1.00
% of variance	25.44	18.13	7.71	5.30	4.37	4.06	3.49	3.19
Cumulative %	25.44	43.57	51.28	56.59	60.96	65.02	68.50	71.69
**Component matrix**								
gallic acid_18	0.052	0.099	−0.135	−0.218	0.199	0.042	0.093	−0.016
3,4-dihydroxybenzoic acid_18	0.054	0.179	0.039	−0.176	0.281	0.091	−0.045	0.086
4-hydroxybenzoic acid_18	0.065	0.299	0.035	−0.112	0.261	−0.051	0.204	−0.179
2,4-dihydroxybenzoic acid_18	0.060	0.262	−0.048	0.003	0.040	−0.102	0.100	0.335
vanillic acid_18	0.067	0.284	0.142	−0.351	0.024	−0.002	0.126	0.044
caffeic acid_18	−0.095	0.123	0.154	0.376	0.342	0.108	−0.252	0.040
syringic acid_18	0.079	0.186	0.328	0.174	0.014	−0.229	−0.096	0.214
p-coumaric acid_18	−0.025	0.247	0.236	0.105	0.291	0.177	−0.077	0.045
ferulic acid_18	0.056	0.296	0.031	0.212	−0.080	0.058	0.118	−0.419
sinapic acid_18	−0.011	0.115	−0.102	0.519	−0.038	0.018	0.295	−0.259
rutin_18	0.085	0.285	−0.215	0.109	−0.309	−0.028	−0.041	0.280
quercetin-3-β-D-glucoside_18	0.041	0.283	−0.199	−0.187	−0.210	0.037	−0.129	−0.012
benzoic acid_18	0.113	0.282	0.175	0.000	0.048	0.030	0.095	0.018
kaempferol-3-O-β-rutinoside_18	0.039	0.323	−0.186	0.171	−0.228	−0.054	−0.129	0.153
quercetin_18	−0.149	0.201	−0.061	−0.030	−0.259	0.319	−0.185	−0.160
cinnamic acid_18	0.026	0.139	−0.056	−0.078	−0.169	0.073	−0.046	−0.351
kaempferol_18	0.020	0.212	0.263	−0.166	0.018	−0.055	−0.291	−0.060
gallic acid_19	−0.004	−0.025	−0.036	−0.014	−0.049	0.104	0.122	0.280
3,4-dihydroxybenzoic acid_19	0.185	−0.073	−0.144	0.000	0.106	0.069	0.152	0.058
4-hydroxybenzoic acid_19	0.261	0.051	−0.261	0.037	0.173	−0.119	0.149	0.130
2,4-dihydroxybenzoic acid_19	0.249	−0.035	0.083	−0.015	−0.070	−0.087	0.091	0.099
vanillic acid_19	0.234	0.043	−0.080	−0.070	0.165	−0.121	0.349	−0.225
caffeic acid_19	0.115	−0.044	0.421	−0.005	−0.340	0.040	0.182	0.025
syringic acid_19	0.178	−0.065	0.303	−0.047	−0.225	0.124	0.291	−0.036
p-coumaric acid_19	0.251	−0.059	0.284	−0.050	−0.160	−0.066	−0.068	0.024
ferulic acid_19	0.309	−0.075	−0.016	0.025	0.049	−0.077	−0.074	−0.069
sinapic acid_19	0.109	−0.057	0.101	0.355	0.058	0.072	0.002	0.094
rutin_19	0.306	−0.026	−0.089	0.133	−0.073	−0.090	−0.144	0.040
quercetin-3-β-D-glucoside_19	0.265	0.032	−0.219	−0.055	−0.003	0.006	−0.198	−0.059
benzoic acid_19	0.326	−0.063	−0.042	−0.010	0.022	0.042	−0.163	−0.066
kaempferol-3-O-β-rutinoside_19	0.325	−0.056	−0.036	0.124	−0.047	−0.002	−0.114	−0.006
quercetin_19	0.252	−0.085	0.029	−0.059	0.037	0.322	−0.274	−0.198
cinnamic acid_19	0.201	−0.107	0.043	−0.004	0.169	0.246	−0.147	0.022
kaempferol_19	0.050	0.009	−0.069	0.016	−0.019	0.709	0.251	0.274

PC: principal component.

**Table 4:** Average cluster values of 17 polyphenol contents of 9 *Amaranthus* species.

Phenolic Compounds	Group 1	Group 2	Group 3	Group 4	Group 5	Group 6
**Hydroxybenzoic acid (** **μg g^−1^)**						
HBA1	1.7 ± 4.6 a ^1^	1.2 ± 1.9 bc	2.4 ± 3.3 a	4.2 ± 3.7 d	4.0 ± 5.7 ab	4.1 ± 3.6 cd
HBA2	5.1 ± 4.0 c	11.4 ± 7.2 b	8.3 ± 6.5 a	1.8 ± 2.0 c	6.5 ± 6.7 bc	1.9 ± 3.0 bc
HBA3	27.4 ± 33.9 b	64.2 ± 58.8 b	56.0 ± 50.1 a	7.3 ± 4.9 b	22.0 ± 22.0 b	25.0 ± 25.0 b
HBA4	31.2 ± 18.3 b	26.1 ± 16.7 b	37.8 ± 28.3 a	7.5 ± 6.6 b	22.3 ± 16.1 b	19.1 ± 13.8 b
HBA5	29.6 ± 13.9 b	36.7 ± 23.0 bc	40.3 ± 33.4 a	9.8 ± 7.4 d	22.3 ± 13.7 bc	17.1 ± 17.4 cd
HBA6	15.7 ± 11.6 a	7.1 ± 6.9 b	7.5 ± 5.6 a	3.3 ± 2.1 b	7.8 ± 7.2 b	5.1 ± 3.4 b
HBA7	115.2 ± 50.0 b	133.9 ± 84.2 bc	98.0 ± 51.9 a	34.4 ± 23.0 d	60.0 ± 31.2 cd	52.8 ± 22.2 cd
**Hydroxycinnamic acid (** **μg g^−1^)**						
HCA1	16.4 ± 15.0 b	8.1 ± 6.8 b	10.2 ± 13.4 a	10.6 ± 9.4 ab	10.0 ± 10.3 b	10.03 ± 5.9 ab
HCA2	16.8 ± 12.1 b	11.3 ± 7.5 bc	11.8 ± 10.3 a	5.6 ± 4.1 bc	7.3 ± 5.8 c	5.8 ± 2.5 bc
HCA3	28.5 ± 9.2 b	32.3 ± 21.5 bc	25.9 ± 9.9 a	12.3 ± 8.7 c	18.2 ± 8.9 c	20.1 ± 8.6 b
HCA4	25.6 ± 11.7 bc	25.6 ± 15.8 c	27.6 ± 17.6 ab	10.4 ± 15.7 c	17.5 ± 14.7 c	32.8 ± 30.5 a
HCA5	3.7 ± 2.4 a	4.5 ± 3.8 a	2.6 ± 2.2 a	0.9 ± 1.2 a	1.6 ± 1.3 a	2.1 ± 1.0 a
**Flavonoid (** **μg g^−1^)**						
FLA1	1931.1 ± 1483.9 b	2115.2 ± 1579.9 bc	1785.0 ± 812.5 a	553.5 ± 535.4 d	979.7 ± 788.4 cd	1609.9 ± 599.1 a
FLA2	108.5 ± 78.9 b	221.0 ± 174.1 b	191.0 ± 107.9 a	43.6 ± 49.5 b	93.0 ± 98.3 b	154.7 ± 109.5 a
FLA3	103.6 ± 73.3 b	112.6 ± 77.8 b	92.3 ± 37.7 a	41.9 ± 23.9 b	53.0 ± 33.2 b	79.7 ± 24.3 a
FLA4	36.7 ± 27.2 cd	41.6 ± 34.5 d	23.7 ± 16.5 bc	24.8 ± 4.9 ab	17.4 ± 11.1 d	29.4 ± 8.5 a
FLA5	9.3 ± 7.2 a	9.9 ± 11.6 b	10.7 ± 8.0 a	8.8 ± 6.2 b	7.0 ± 6.5 b	9.3 ± 6.4 b

^1^ The same letter in each column indicates no significant difference per Duncan’s multiple range test, *p* < 0.05. HBA1: gallic acid; HBA2: 3,4-dihydroxybenzoic acid; HBA3: 4-hydroxybenzoic acid; HBA4: 2,4-dihydroxybenzic acid; HBA5: vanillic acid; HBA6: syringic acid; HBA7: benzoic acid; HCA1: caffeic acid; HCA2: p-coumaric acid; HCA3: ferulic acid; HCA4: sinapic acid; HCA5: cinnamic acid; FLA1: rutin; FLA2: quercetin-3-β-D-glucoside; FLA3: kaempferol-3-O-β-rutinoside; FLA4: quercetin; FLA5: kaempferol.

**Table 5:** Exploring the differential impact of genotype and year on polyphenol variability.

Type of Polyphenol	Genotype	Year	Genotype × Year
gallic acid	1.958 ***	8.907 ***	1.484 ***
3,4-dihydroxybenzoic acid	10.93 ***	9.652 ***	6.756 ***
4-hydroxybenzoic acid	326.4 ***	2996.4 ***	293.1 ***
2,4-dihydroxybenzic acid	78.37 ***	98.77 ***	41.32 ***
vanillic acid	104.8 ***	356.7 ***	63.9 ***
syringic acid	9.53 ***	45.32 ***	7.18 ***
benzoic acid	910.6 ***	2483.8 ***	686.2 ***
caffeic acid	6.228 ***	14.131 ***	18.443 ***
p-coumaric acid	9.933 ***	17.154 ***	17.368 ***
ferulic acid	34.32 ***	45.07 ***	39.23 ***
sinapic acid	102.93 ***	30.22 *	33.32 ***
cinnamic acid	0.885 ***	3.742 ***	1.163 ***
rutin	221,815 ***	1,656,986 ***	168,219 ***
quercetin-3-β-D-glucoside	2878 ***	17,369 ***	1785 ***
kaempferol-3-O-β-rutinoside	431 ***	3445 ***	532 ***
quercetin	13 ***	716.8 ***	72 ***
kaempferol	0.71 *	222.69 ***	1.17 ***

*: *p* < 0.01 and ***: *p* < 0.001.

## Data Availability

Data are contained within the article and Supplementary Materials.

## Supplementary Materials

The following supporting information can be downloaded at: https://www.mdpi.com/article/10.3390/antiox13040501/s1, Table S1: List of 120 Amaranthus accessions used in this study; Table S2: The UPLC-PDA analysis of polyphenol content among 120 Amaranthus accessions between the year of 2018 and 2019; Table S3: Relative polyphenol content index of 120 Amaranthus accessions between the year 2018 and 2019; Table S4: Correlations analysis between 17 polyphenols in 9 Amaranthus species between the year of 2018 and 2019.
